# Supplementary material for: Theoretical step approach with ‘Three-pillar’ device assistance for successful endoscopic transpapillary gallbladder drainage
Source: PLoS One. 2023 Feb 9;18(2):e0281605. doi: 10.1371/journal.pone.0281605 (PMC9910654; doi:10.1371/journal.pone.0281605)
Supplement: S1 Appendix — (DOCX) [file pone.0281605.s001.docx]

**S1 Appendix**

**Lists of the patients recruited in this study compared to the previous publication**

| Classical ETGBD | |  |  | Strategic ETGBD | |  |  |  |
| --- | --- | --- | --- | --- | --- | --- | --- | --- |
| This study | Ref. 16 |  |  | This study | Ref. 16 | Ref. 17 | Ref. 18 | Ref. 19 |
| Case No. |  |  |  | Case No. |  |  |  |  |
| **#1** | **x** |  |  | **#1** | **x** |  |  |  |
| ⁝ | ⁝ |  |  | ⁝ | ⁝ |  |  |  |
| **#50** | **x** |  |  | **#10** | **x** |  | **x** | **x** |
|  |  |  |  | ⁝ | ⁝ |  |  |  |
|  |  |  |  | **#16** | **x** |  |  | **x** |
|  |  |  |  | **#17** | **x** |  |  |  |
|  |  |  |  | **#18** | **x** |  |  | **x** |
|  |  |  |  | **#19** | **x** |  |  |  |
|  |  |  |  | **#20** | **x** | **x** |  |  |
|  |  |  |  | ⁚ | ⁚ |  |  |  |
|  |  |  |  | **#23** | **x** |  |  | **x** |
|  |  |  |  | **#24** | **x** |  |  | **x** |
|  |  |  |  | **#25** | **x** | **x** |  |  |
|  |  |  |  | ⁝ | ⁝ |  |  |  |
|  |  |  |  | **#46** | **x** |  |  |  |
|  |  |  |  | ⁝ |  |  |  |  |
|  |  |  |  | **#51** |  |  |  | **x** |
|  |  |  |  | ⁚ |  |  |  |  |
|  |  |  |  | **#54** |  |  |  | **x** |
|  |  |  |  | **#55** |  |  |  | **x** |
|  |  |  |  | **#56** |  |  |  | **x** |
|  |  |  |  | ⁝ |  |  |  |  |
|  |  |  |  | **#65** |  |  |  |  |

Ref. 16, Yoshida M. Gut and Liver 2021; Ref. 17, Miyabe K. Endoscopy 2019; Ref. 18, Yoshida M. Endoscopy 2019; Ref. 19, Yoshida M. Digestive Endoscopy 2022.
