# Supplementary material for: Theoretical step approach with ‘Three-pillar’ device assistance for successful endoscopic transpapillary gallbladder drainage
Source: PLoS One. 2023 Feb 9;18(2):e0281605. doi: 10.1371/journal.pone.0281605 (PMC9910654; doi:10.1371/journal.pone.0281605)
Supplement: S1 Table — (DOCX) [file pone.0281605.s002.docx]

**S1 Table.**

**The correlation of the four-step classification to the severity of grade of AC**

| Four-step classification | Severity grade of AC | N |  | Classical | | Strategic | | P-value |
| --- | --- | --- | --- | --- | --- | --- | --- | --- |
| Category 0 | Severe | 1 |  | 1 | (7.1%) | 0 | (0%) | N/A |
| Category 1 | Mild | 7 |  | 3 | (21.4%) | 4 | (11.4%) |  |
|  | Moderate | 10 |  | 3 | (21.4%) | 7 | (20.0%) | 0.859 |
|  | Severe | 3 |  | 1 | (7.1%) | 2 | (5.7%) |  |
| Category 2 | Mild | 2 |  | 1 | (7.1%) | 1 | (2.9%) | 0.386 |
|  | Moderate | 1 |  | 0 | (0%) | 1 | (2.9%) |  |
| Category 3a | Moderate | 3 |  | 1 | (7.1%) | 2 | (5.7%) | N/A |
| Category 3b | Mild | 5 |  | 1 | (7.1%) | 4 | (11.4%) |  |
|  | Moderate | 13 |  | 2 | (14.3%) | 11 | (31.4%) | 0.880 |
|  | Severe | 1 |  | 0 | (0%) | 1 | (2.9%) |  |
| Category 4 | Mild | 1 |  | 1 | (7.1%) | 0 | (0%) | 0.083 |
|  | Moderate | 2 |  | 0 | (0%) | 2 | (5.7%) |  |

AC, acute cholecystitis; N/A, not applicable.
