# Supplementary material for: Theoretical step approach with ‘Three-pillar’ device assistance for successful endoscopic transpapillary gallbladder drainage
Source: PLoS One. 2023 Feb 9;18(2):e0281605. doi: 10.1371/journal.pone.0281605 (PMC9910654; doi:10.1371/journal.pone.0281605)
Supplement: S2 Table — (DOCX) [file pone.0281605.s003.docx]

**S2 Table.**

**The correlation of the severity of grade of AC to the technical success rate**

| Severity grade of AC | Classical ETGBD | |  | Strategic ETGBD | |  | P-value |
| --- | --- | --- | --- | --- | --- | --- | --- |
|  | Success | Failure | Technical Success rate | Success | Failure | Technical Success rate |  |
| Mild | 25 | 6 | 80.6% | 34 | 0 | 100% | 0.009 |
| Moderate | 6 | 6 | 50% | 24 | 2 | 92.3% | 0.007 |
| Severe | 5 | 2 | 71.4% | 5 | 0 | 100% | 0.318 |

AC, acute cholecystitis; N/A, not applicable.
