# Supplementary material for: Theoretical step approach with ‘Three-pillar’ device assistance for successful endoscopic transpapillary gallbladder drainage
Source: PLoS One. 2023 Feb 9;18(2):e0281605. doi: 10.1371/journal.pone.0281605 (PMC9910654; doi:10.1371/journal.pone.0281605)
Supplement: S3 Table — (DOCX) [file pone.0281605.s004.docx]

**S3 Table.**

**Overall procedure time**

|  | Number | Overall procedure time (median, min) | (range) | P-value |
| --- | --- | --- | --- | --- |
| Classical ETGBD | 50 | 44.5 | (10-142) | 0.35 |
| Strategic ETGBD | 65 | 51 | (13-147) |  |
|  |  |  |  |  |
| Failed cases in Classical ETGBD | 14 | 52.5 | (35-98) | 0.69 |
| Failed cases in Strategic ETGBD | 2 | 65.5 | (61-70) |  |

ETGBD, endoscopic transpapillary gallbladder drainage
