# Supplementary figures and images for: Theoretical step approach with ‘Three-pillar’ device assistance for successful endoscopic transpapillary gallbladder drainage
Source: PLoS One. 2023 Feb 9;18(2):e0281605. doi: 10.1371/journal.pone.0281605 (PMC9910654; doi:10.1371/journal.pone.0281605)

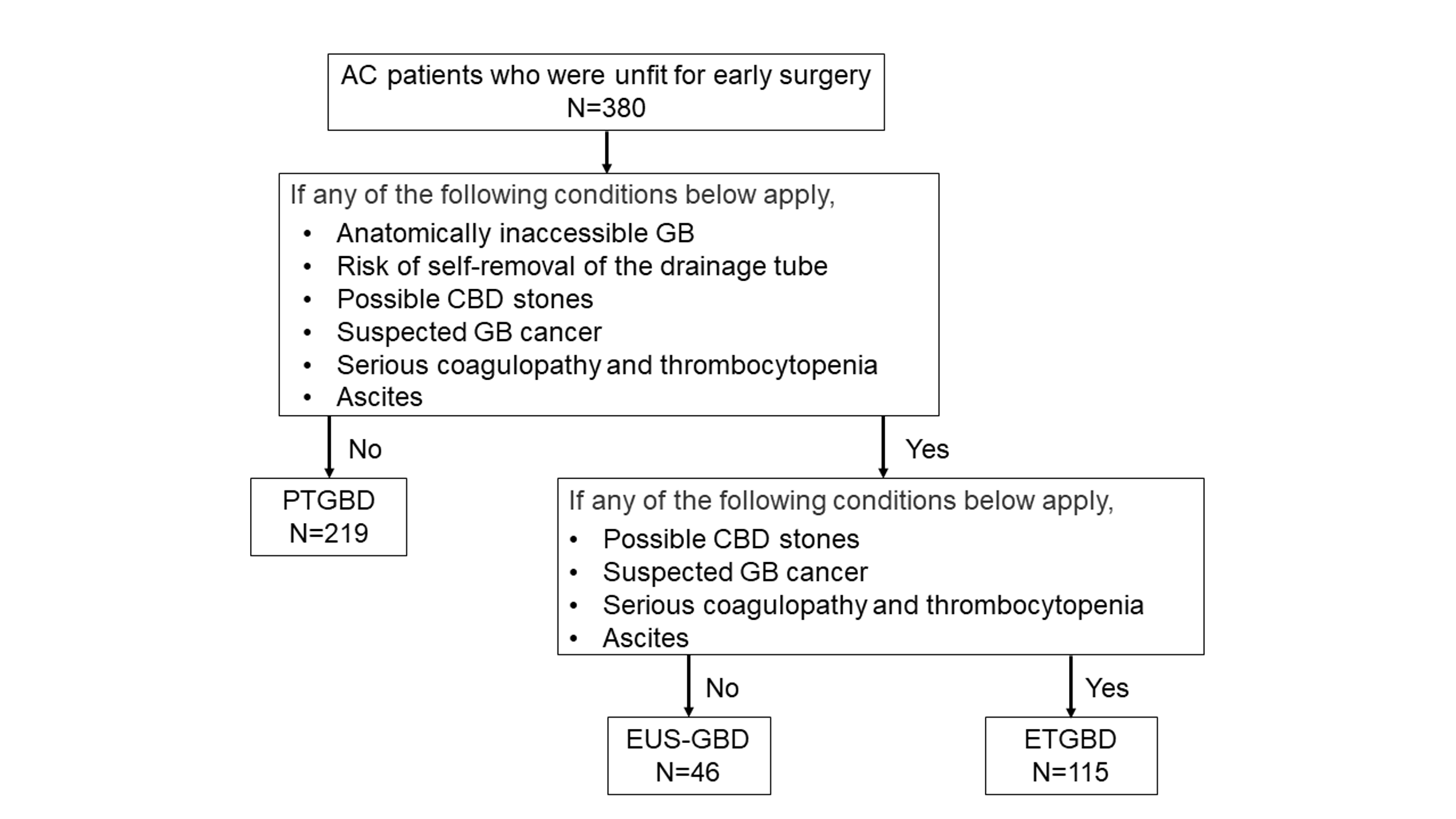

Supplement: S1 Fig — In our practical applications during this period, fundamental strategies for the patients of acute cholecystitis (AC) in whom are unfit for early surgery (N = 380) are as follows:1) Percutaneous transhepatic gallbladder drainage (PTGBD) is the first-line drainage procedure (N = 219). 2) When PTGBD cannot be performed in patients with an anatomically inaccessible GB, those at risk of self-removal of the drainage tube, endoscopic ultrasound-guided GB drainage (EUS-GBD) (N = 46) or endoscopic transpapillary GB drainage (ETGBD) (N = 115) is the second-line drainage procedure. 3) In patients with possible common bile duct (CBD) stones, suspected GB cancer, serious coagulopathy and thrombocytopenia, or ascites, ETGBD is preferentially performed as the alternative technique. (TIF) [file pone.0281605.s005.tif]
