# Supplementary material for: Theoretical step approach with ‘Three-pillar’ device assistance for successful endoscopic transpapillary gallbladder drainage
Source: PLoS One. 2023 Feb 9;18(2):e0281605. doi: 10.1371/journal.pone.0281605 (PMC9910654; doi:10.1371/journal.pone.0281605)
Supplement: Attachment — Submitted filename: Response to Reviewers R2.docx [file pone.0281605.s007.docx]

December 24, 2022

Dear Professor Alejandro Piscoya, Academic Editor

*PLOS ONE*

Professor Alejandro Piscoya,

We are pleased that you are interested in our paper, and reviewer’s comments are helpful for our manuscript to become a more substantial paper.

We have answered all reviewer’s comments adequately. We highlighted the changes to the revised manuscript by colored text ‘Revised Manuscript with Track Changes R2’. A point-by-point reply to the Journal Requirements and the reviewer’s comments is as follows.

Journal Requirements:

Please review your reference list to ensure that it is complete and correct. If you have cited papers that have been retracted, please include the rationale for doing so in the manuscript text, or remove these references and replace them with relevant current references. Any changes to the reference list should be mentioned in the rebuttal letter that accompanies your revised manuscript. If you need to cite a retracted article, indicate the article’s retracted status in the References list and also include a citation and full reference for the retraction notice.

We reviewed our reference list to ensure that it is complete and correct.

Reviewer #2: It was well revised and written, but there were some point to be clarified.

1. Though this study is a retrospective nature, it would be important to show overall procedure time in both groups.

As suggested, we added overall procedure time of both groups in the ‘Procedure time’ part (Results part) and S3 table. Thank you for the suggestion.

2. Please provide overall procedure time in failed cases in both groups to minimize the potential selection biases.

As suggested, we also added overall procedure time of failed cases in the ‘Procedure time’ part (Results part) and S3 table. Thank you for the suggestion.

3. In the study periods, how many patients underwent gallbladder drainage other than ETGBD. Please provide the flow chart of the management of acute cholecystitis during this periods.

In our practical applications during this period, fundamental strategies for the patients of AC in whom are unfit for early surgery (N=380) are as follows:1) PTGBD is the first-line drainage procedure (N=219). 2) When PTGBD cannot be performed in patients with an anatomically inaccessible GB, those at risk of self-removal of the drainage tube, EUS-GBD (N=46) or ETGBD (N=115) is performed as the second-line drainage procedure. 3) In patients with possible CBD stones, suspected GB cancer, serious coagulopathy and thrombocytopenia, or ascites, ETGBD is preferentially performed.

We updated these explanations of the strategy in the Discussion part, and added the flow chart of the management of AC during this period in S1 Figure.

Thank you for the suggestion.

Thank you very much for your consideration of our paper. We look forward to hearing from you again.

Sincerely yours,

Itaru Naitoh

Itaru Naitoh, MD, PhD.

Department of Gastroenterology and Metabolism

Nagoya City University Graduate School of Medical Sciences, Nagoya, Japan

1 Kawasumi, Mizuho-cho, Mizuho-ku Nagoya 467-8601, Japan

Phone: +81-52-853-8211, Fax: +81-52-852-0952.

e-mail: inaito@med.nagoya-cu.ac.jp
